# A Mild Form of *SLC29A3* Disorder: A Frameshift Deletion Leads to the Paradoxical Translation of an Otherwise Noncoding mRNA Splice Variant

**DOI:** 10.1371/journal.pone.0029708

**Published:** 2012-01-04

**Authors:** Alexandre Bolze, Avinash Abhyankar, Audrey V. Grant, Bhavi Patel, Ruchi Yadav, Minji Byun, Daniel Caillez, Jean-Francois Emile, Marçal Pastor-Anglada, Laurent Abel, Anne Puel, Rajgopal Govindarajan, Loic de Pontual, Jean-Laurent Casanova

**Affiliations:** 1 St. Giles Laboratory of Human Genetics of Infectious Diseases, Rockefeller Branch, The Rockefeller University, New York, New York, United States of America; 2 Laboratory of Human Genetics of Infectious Diseases, Necker Branch, Institut National de la Santé et de la Recherche Medicale, INSERM U980, University Paris Descartes, Paris, France; 3 Wilson Pharmacy, Department of Pharmaceutical and Biomedical Sciences, University of Georgia, Athens, Georgia, United States of America; 4 Laboratory of Pathology, Groupe Hospitalier du Havre, Jacques Monod Hospital, Montivilliers, France; 5 EA4340, University Versailles SQY and Ambroise Paré Hospital, APHP, Boulogne, France; 6 Department of Biochemistry and Molecular Biology, Institute of Biomedicine (IBUB), University of Barcelona and National Institute for the Study of Liver and Gastrointestinal Diseases (CIBER EHD), Barcelona, Spain; 7 Department of Pediatrics, Jean Verdier Hospital, APHP, University Paris 13, Bondy, France; 8 Pediatric Immunology-Hematology Unit, Necker Hospital for Sick Children, Paris, France; University of Bonn, Institut of Experimental Hematology and Transfusion Medicine, Germany

## Abstract

We investigated two siblings with granulomatous histiocytosis prominent in the nasal area, mimicking rhinoscleroma and Rosai-Dorfman syndrome. Genome-wide linkage analysis and whole-exome sequencing identified a homozygous frameshift deletion in *SLC29A3*, which encodes human equilibrative nucleoside transporter-3 (hENT3). Germline mutations in *SLC29A3* have been reported in rare patients with a wide range of overlapping clinical features and inherited disorders including H syndrome, pigmented hypertrichosis with insulin-dependent diabetes, and Faisalabad histiocytosis. With the exception of insulin-dependent diabetes and mild finger and toe contractures in one sibling, the two patients with nasal granulomatous histiocytosis studied here displayed none of the many *SLC29A3*-associated phenotypes. This mild clinical phenotype probably results from a remarkable genetic mechanism. The *SLC29A3* frameshift deletion prevents the expression of the normally coding transcripts. It instead leads to the translation, expression, and function of an otherwise noncoding, out-of-frame mRNA splice variant lacking exon 3 that is eliminated by nonsense-mediated mRNA decay (NMD) in healthy individuals. The mutated isoform differs from the wild-type hENT3 by the modification of 20 residues in exon 2 and the removal of another 28 amino acids in exon 3, which include the second transmembrane domain. As a result, this new isoform displays some functional activity. This mechanism probably accounts for the narrow and mild clinical phenotype of the patients. This study highlights the ‘rescue’ role played by a normally noncoding mRNA splice variant of *SLC29A3*, uncovering a new mechanism by which frameshift mutations can be hypomorphic.

## Introduction

**Figure 1:**
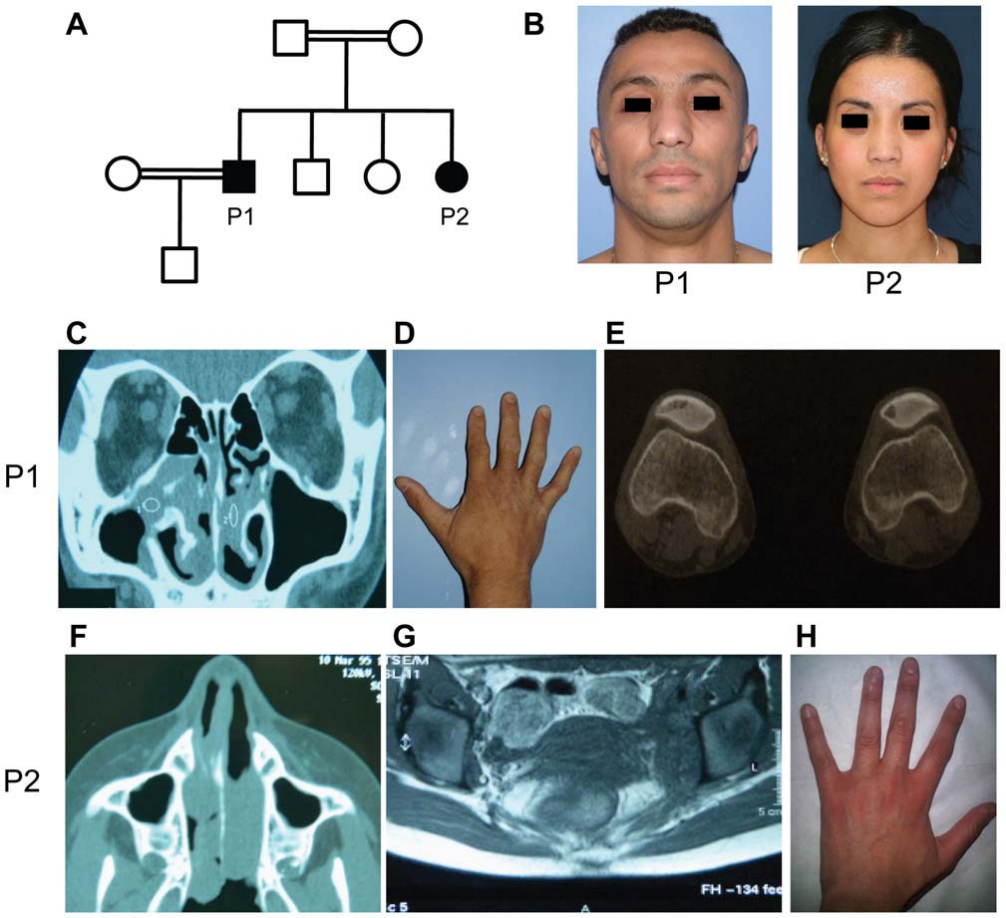
Clinical and radiological data of the patients P1 and P2. (A) Pedigree of the family. Squares: male subjects; circles: female subjects; closed symbols: affected individuals; open symbols: unaffected individuals; double horizontal line: consanguineous marriage. (B) Both patients suffer from chronic nasal obstruction, with enlarged noses. (C–E) Clinical phenotypes of P1. (C) CT scan of the sinuses showing a nasal mass. (D) Mild finger contractures with campodactyly. (E) Lytic lesions of both patellae. (F–H) Clinical phenotype of P2. (F) CT scan of the sinuses showing a nasal mass. (G) Abdominal CT scan with retroperitoneal fibrosis. (H) No hand abnormalities.

Rhinoscleroma (RS) is a chronic, granulomatous disease of the upper respiratory tract caused by the bacteria *Klebsiella rhinoscleromatis.* Migration from an area in which RS is endemic (such as North Africa or Central America) appears to be an important factor in disease development, consistent with infection occurring during childhood [1]. However, RS is rare and it is possible that only a small fraction of infected individuals develop the disease, suggesting that RS may also result from host predisposition due to an inherited or acquired immunodeficiency [1]. The recent discoveries of genetic etiologies of infectious diseases of childhood as diverse as tuberculosis [2], herpes simplex encephalitis [3], invasive pneumococcal disease [4], and chronic mucocutaneous candidiasis [5], [6] suggest that single-gene inborn errors of immunity may also underlie other life-threatening infectious diseases of childhood, including RS [7], [8]. We describe here the investigation of two patients born to a consanguineous family from Morocco (Figure 1A). Both patients were originally diagnosed with RS (individuals 7 and 8, family VII in [1]) at the ages of 5 and 14 years, with no overt acquired immunodeficiency. We therefore hypothesized that RS may segregate as an autosomal recessive trait in this kindred.

## Results

### Case reports

Two siblings were originally diagnosed with RS on the basis of nasal obstruction, epistaxis, a granulomatous appearance of the nasal mucosa and radiological and histological analyses [1]. However, important new data were recently obtained, leading to a revision of this diagnosis, as explained below.

The older brother, P1 (Figure 1A, B), is the first child of first-cousin parents of Moroccan origin. He was born at term, with normal health indicators and metrics, following an uneventful pregnancy. P1 has an adult height of 162 cm, which is close to 167 cm, his predicted adult height based on the heights of his parents (his father is 168 cm tall and his mother is 155 cm tall). He was diagnosed with insulin-dependent diabetes at the age of 5 years. He was subsequently diagnosed with exocrine pancreas insufficiency at the age of 9 years, following the observation of bulky stools and steatorrhea with levels of fat in stool at 12 g/24 hr. At this stage, a diagnosis of cystic fibrosis was excluded and a computed tomography (CT) scan of the pancreas was normal. At the age of 14 years, nasal obstruction occurred, with infiltrates found in both the nose and the right maxillary sinus (Figure 1C). P1 was then diagnosed with RS. Several operations were required to free the nasal fossae, between the ages of 14 and 18 years. An improvement was observed after the initiation of antibiotic treatment, but multiple relapses occurred 10 months after treatment, despite the use of various combinations of antibiotics. Apparently spontaneous recovery was observed from the age of 18 years onwards. Biological signs of inflammation persisted, with both a high erythrocyte sedimentation rate (>50 mm) and polyclonal hypergammaglobulinemia (IgG = 15.3 g/l, IgA = 4.2 g/l, IgM = 2.8 g/l). Finally, P1 developed a very mild contracture of the fingers and toes at the age of 12 years (Figure 1D). At last follow up, the patient was 33 years old and had insulin-dependent diabetes and exocrine pancreatic deficiency. He is doing well, with daily subcutaneous insulin and oral pancreatic extract treatments, which have normalized his glycemia and steatorrhea. He has required no further surgery since the age of 18 years, but still suffers from chronic nasal obstruction and an enlarged nose due to the presence of a nasal mass (Figure 1B). He recently suffered from local pain of the knee. X-ray revealed lytic bone lesions of both patellas (Figure 1E). No other lesions were detected on X-ray or bone scintigraphy. This patient is otherwise healthy (Table 1). Both cardiac morphology and function were normal on cardiac ultrasonography (US). Dermatologist examination revealed no pigmented hypertrichotic skin lesions and skin biopsies taken at 3 different sites were normal. Speech and tone audiometries were normal. Ophtalmologic examination revealed mild diabetic retinopathy but no uveitis and no histiocytic infiltration.

**Table 1:** Clinical phenotype of the patients and patients already reported in the literature with SLC29A3 mutations.

	P1	P2	H syndrome	PHID	FHC	SHML	Mild SLC29A3 disorder
Nasal infiltration	+	+	−	−	+	+	−
Hyperpigmentation	−	−	+	+	−	−	−
Hypertrichosis	−	−	+	+	−	−	−
Sensorineural deafness	−	−	+	−	+	+	+
Hepatomegaly	−	−	+	+	−	+	−
Short stature	−	−	+	+	+	+	−
Hypogonadism	−	−	+	−	+	−	−
Gynecomastia	−	−	+	−	−	−	−
Delayed puberty	−	−	−	+	−	−	−
Pancreatic exocrine deficiency	+	−	−	+	−	−	−
Insulin-dependent diabetes	+	−	−	+	−	−	−
Uveitis	−	−	+	−	+	+	−
Hallux valgus	−	−	+	−	−	−	−
Contractures of the fingers	+	−	+	−	+	−	−
Contractures of the toes	+	−	+	−	+	−	−
Contracture of the ankle	−	−	−	−	+	−	−
Myelofibrosis	−	−	+	−	−	+	−
Retroperitoneal fibrosis	−	+	−	−	−	−	−
Lymphadenopathy	−	−	−	+	+	+	+
Heart anomalies	−	−	+	−	−	−	−
References	this report	this report	19	16	20	20	23

The second patient, P2 (Figure 1A, B), is the fourth and youngest sibling in the family. At the age of five years, she developed a nasal obstruction and epistaxis (Figure 1F). Her condition initially seemed to improve on antibiotic treatment, but she had relapses at the ages of six and seven years, necessitating surgery to free the nasal fossae. Like her brother, she displayed chronic inflammation, with a high erythrocyte sedimentation rate (>80 mm) and polyclonal hypergammaglobulinemia (IgG = 16.2 g/l, IgA = 5.1 g/l, IgM = 3.1 g/l). At the age of 17 years, she presented with retroperitoneal fibrosis (Figure 1G). At her last follow up, the patient was 23 years old with an adult height of 162 cm. She did not display any signs of insulin-dependent diabetes or exocrine pancreatic deficiency and is otherwise healthy (Figure 1H and Table 1). Both cardiac morphology and function were normal on cardiac US. Dermatologist examination revealed no pigmented hypertrichotic skin lesions. Speech and tone audiometries were normal. Ophtalmologic examination was normal with no uveitis and no histiocytic infiltration. All these clinical results and the comparisons with patients reported with mutations in *SLC29A3* are reported in Table 1.

### Histological and microbiological features

**Figure 2:**
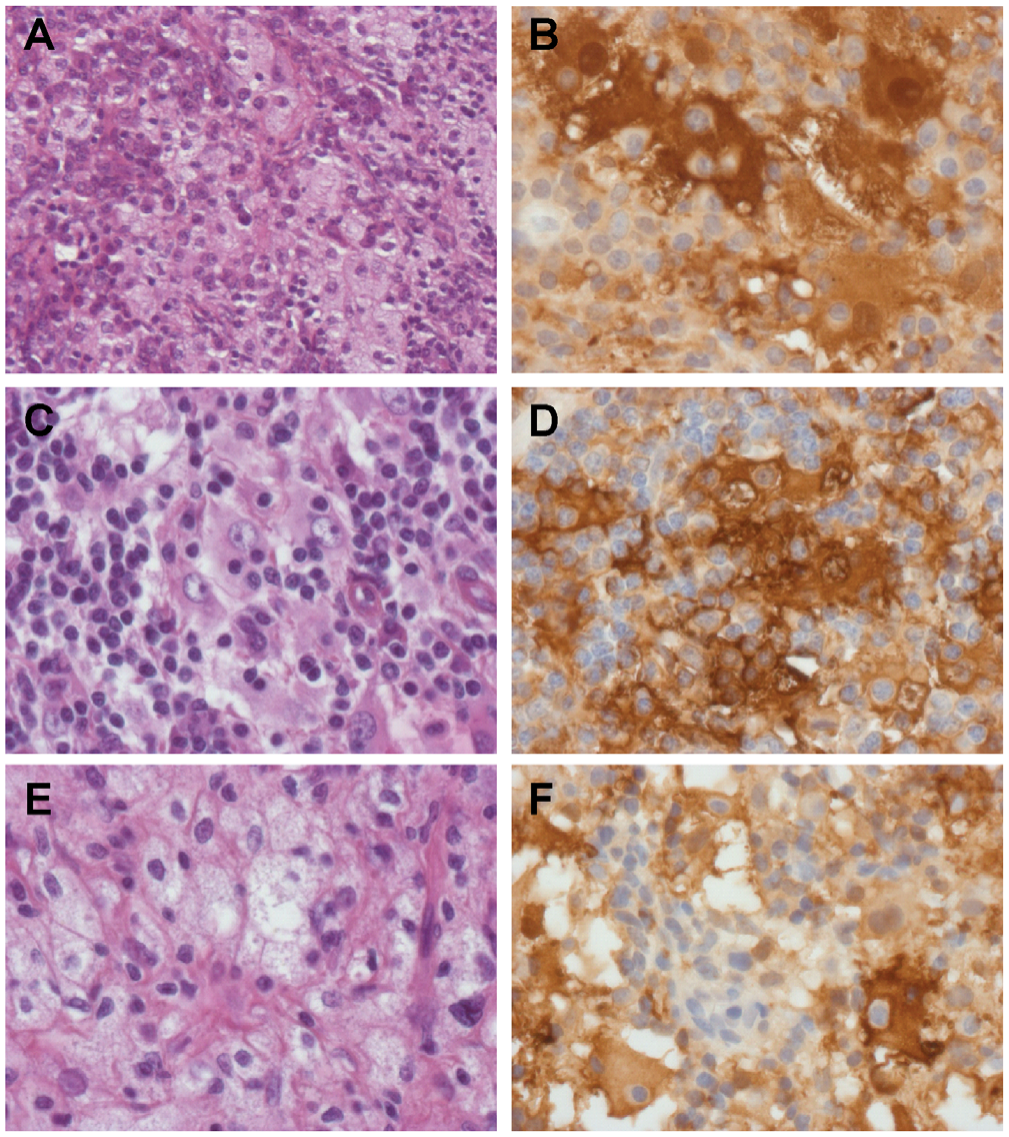
Histology of nasal (A, B, F) and skin (C, D, E) biopsy specimens from P1 (A–E) and P2 (F). Most biopsy specimens contained vacuolated histiocytes suggestive of Mikulicz cells (A, E). Some specimens also contained large S100 protein-positive histiocytes enclosing lymphocytes (emperipolesis) (B, C, D, F). H&E staining (A, C, E) and immunohistochemical staining for S100 protein (B, D, F). Original magnification ×200 (A) and ×400 (B–F).

RS was diagnosed partly on the basis of the histological findings for the two patients. Slides of several biopsy specimens were recently reviewed, with additional immunohistochemical analysis. For P1, specimens from nasal cavity biopsies performed at the ages of 18 and 30 years were available for histological review. On the first biopsy, the mucosa displayed fibrosis and a mild to moderate inflammatory infiltrate containing plasma cells, lymphocytes and some vacuolated histiocytes. The histiocytes resembled Mikulicz cells. However Wartin-Starry, Grocott and periodic acid-Schiff (PAS) stains were negative. The nasal biopsy specimen obtained at the age of 30 years also contained areas with vacuolated histiocytes and numerous plasma cells (Figure 2A). In addition, other parts of the biopsy specimens also displayed specific features of Rosai-Dorfman histiocytosis, as they contained several large histiocytes with emperipolesis. These histiocytes were positive for CD68, CD4 and S100 protein (Figure 2B). P1 also underwent resection of a large (2 cm diameter) subcutaneous nodule of the right arm at the age of 19 years. This nodule was well circumscribed and localized within the deep dermis and hypodermis. A diagnosis of Rosai-Dorfman histiocytosis was clear, based on the presence of several large S100 protein-positive histiocytes with emperipolesis (Figure 2C–D). Most of the cells contained within macrophages were CD4 lymphocytes. Mononucleated vacuolated and spumous macrophages were also abundant (Figure 2E).

For P2, nasal cavity biopsies performed at the age of 12 years disclosed histological lesions similar to those of the second nasal sample from P1, typical of Rosai-Dorfman histiocytosis. The histiocytes with emperipolesis were positive for CD68, CD4 and S100 protein (Figure 2F). CD1a and factor XIIIa staining were not conclusive, probably due to the use of Bouin fixation. A biopsy specimen from the area of retroperitoneal fibrosis displayed fibrosis and the presence of a few mononucleated cells, but no vacuolated or spumous histiocytes or emperipolesis.

In summary, the nasal cavity biopsy specimens of both siblings presented histological lesions of chronic inflammation, with fibrosis, large numbers of plasma cells and vacuolated histiocytes mimicking Mikulicz cells. However, some nasal biopsy specimens and one skin nodule also contained large S100 protein-positive histiocytes with lymphocyte emperipolesis typical of Rosai-Dorfman histiocytosis. It should also be stressed that bacterial cultures of *Klebsiella Rhinoscleromatis* in the nasal biopsy specimens from both patients were negative.

### Genome-wide linkage and whole-exome sequencing

**Figure 3:**
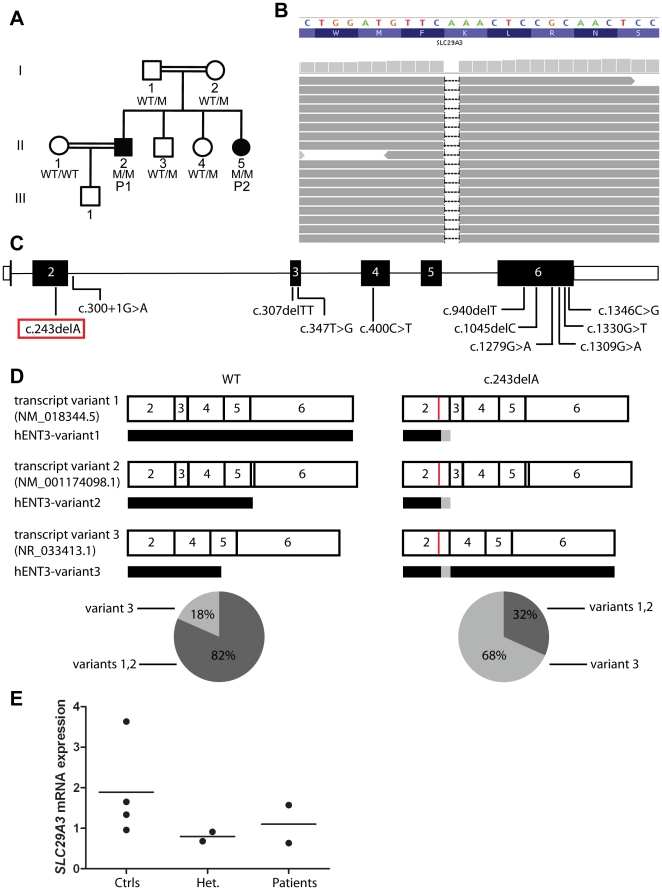
Identification of a frameshift deletion in *SLC29A3*. (A) Pedigree of the family. The *SLC29A3* genotypes of the patients and the family members from whom DNA was available for sequence analysis are listed under their symbols. Genotyping was carried out twice. (B) Illumina sequencing reads displayed for patient P1. Reads overlapping the mutation in exon 2 of *SLC29A3* (bp position g.73,082,741–g.73,082,765; hg19; NCBI 37) show the homozygous deletion of one A leading to a frameshift (c.243delA). (C) Diagram of *SLC29A3.* Introns are represented by a straight line. 5′-UTR and 3′-UTR are represented by open rectangles. The coding region is represented by closed rectangles. Previously reported mutations are indicated at the corresponding locations. The mutation identified in P1 and P2 is shown in a red rectangle. (D–E) Abnormal expression of *SLC29A3* transcript variants 1, 2 and 3 in the patients' EBV-B cells. (D) *SLC29A3* exons 2–4 were amplified from cDNA obtained from the EBV-B cells of two controls (Ctrl 1 and Ctrl 2) and two patients (P1 and P2) and were ligated to the pCR2.1 vector. Clones containing *SLC29A3* transcripts were sequenced, and the frequency of each variant was calculated by dividing the number of clones containing the particular transcript by the total number of sequenced clones. For Ctrl1: variants 1 and 2: 65/81 and variant 3: 16/81. For Ctrl2: variants 1 and 2: 37/44 and variant 3: 7/44. For P1: variants 1 and 2: 31/85 and variant 3: 54/85. For P2: variants 1 and 2: 20/76 and variant 3: 56/76. Each variant is represented by a diagram, with numbers indicating the number of the exon. The red vertical line indicates the position of the c.243delA mutation. The closed rectangle below each transcript variant represents the corresponding translation products. Gray boxes indicate amino acids modified with respect to the WT form. (E) Levels of *SLC29A3* mRNA (variants 1 and 3 combined) were assessed by Q-PCR on EBV-B cells from four healthy controls (Ctrls), two parents (Het.), and the two patients. Threshold cycles (Ct) for *SLC29A3*, normalized with respect to those of GUS (ΔCt), are plotted as 2^−(ΔCt)^. Each dot represents the mean of three independent experiments for each individual. The horizontal bars indicate the mean for all individuals sharing the same genotype.

We searched for morbid lesions by combining genome-wide linkage (GWL) analysis by homozygosity mapping and whole-exome sequencing (WES) by next-generation deep sequencing [9], [10]. This approach has already proved successful in analyses of the first human patients with FADD deficiency in a kindred with bacterial and viral infections [11], and in deciphering the cause of nonsyndromic hearing loss DFNB82 [12]. For the linkage analysis, we genotyped the two affected patients (P1 and P2) and their parents (I.1 and I.2) (Figure 3A) with the Affymetrix 6.0 array. Seven regions (>1 Mb) were homozygous in the two affected patients and heterozygous in other members of the family (Table 2). In parallel, WES of P1 was performed using the Agilent SureSelect Human All Exon 38 Mb kit. We identified a total of 17,687 coding variations after alignment and variant calling. We identified 177 coding or splicing variants in the linked regions (Table S1). Only two of these variants were novel, not being reported in the NCBI dbSNP 134, in the data from the first 1,094 genomes sequenced from the 1000 Genomes Project (June 2011 data release on http://www.1000genomes.org) [13] (Table 2), or our in-house database (composed of more than 200 exomes from patients with different clinical phenotypes). The two variants mapped to the chromosome 10 linkage region. The first is a homozygous deletion of 1 nucleotide, c.241-243delA (referred to hereafter as c.243delA), in exon 2 of *SLC29A3* (NM_018344.5, MIM 612373) (Figure 3B, C), leading to a frameshift starting at amino acid 81, p.K81Nfs (referred to hereafter as 81fs). The second variant identified is a homozygous c.610G>C mutation in exon 5 of *VCL* (NM_003373.3, MIM 193065), leading to the amino acid change valine to leucine at amino acid position 204, p.V204L. We validated these variants by Sanger sequencing on genomic DNA from peripheral blood and on cDNA from Epstein-Barr virus-transformed B cells (EBV-B cells).

**Table 2:** Whole-exome analysis results of P1.

	Total coding^a^	Coding in 1kgenomes	Coding in dbSNP134	Novel coding^a^	Total splice^a^	Novel splice^a^	Total ncRNA^a^	Novel ncRNA^a^
Whole Exome	17,687	15,424	9,999	261	554	13	56	0

Linked regions										
Chr.	Start marker – position^b^	End marker – position^b^	Total coding	Coding in 1kgenomes	Coding in dbSNP134	Novel coding	Total splice	Novel splice	Total ncRNA	Novel ncRNA
1	rs6656194 -165,765,014	rs12120152 -171,723,809	37	37	36	0	3	0	0	0
1	rs6683655 -188,436,602	rs4329563 -202,090,641	49	48	48	0	4	0	0	0
6	rs342658 –124,035,264	rs2136055 –130,966,801	13	13	13	0	0	0	0	0
8	rs2167362 –34,980,598	rs543864 –36,995,907	0	0	0	0	0	0	0	0
10	rs7911065 –63,030,749	rs4980022 –80,834,685	61	58	58	**2**	6	0	0	0
12	rs1387760 –92,177,784	rs2128894 –94,883,246	4	4	4	0	0	0	0	0
22	rs6008817 –46,814,671	rs1884516 –48,325,524	5	5	5	0	0	0	0	0

Coding variants include missense, nonsense, frameshift, in-frame deletions and insertions and readthrough variants.

Splice variants include all variants within 8 bp in the intron side, or 3 bp in the exon side of a splice junction.

a : Both homozygous and heterozygous variations are included.

b : Position coordinates for the markers correspond to the hg19, NCBI build 37.

### Identification of c.243delA in *SLC29A3* as the disease-causing mutation

The two variants identified by WES were considered as potentially disease-causing. However, *in silico* analysis with PolyPhen2 [14] predicted that the c.610G>C variant in *VCL* would be benign. We also checked the mRNA levels of *VCL* in the patients' EBV-B cells and showed that they were similar to those in control cells (data not shown). Finally mutations in *VCL* have been linked to hypertrophic cardiomyopathy [15], a condition not observed in P1 and P2. By contrast, the *SLC29A3* variant led to a frameshift in exon 2 (Figure 3C). In addition to our analyses of the databases described earlier, we also sequenced 70 healthy individuals from Morocco. None of these controls presented the variation, suggesting that it is not an irrelevant polymorphism. This mutation segregated with the disease status in all family members (Figure 3A). Interestingly, various disorders due to *SLC29A3* mutations have only recently been documented, including H syndrome (MIM 612391), pigmented hypertrichosis with insulin-dependent diabetes mellitus (PHID) syndrome (MIM 612391), Faisalabad histiocytosis (FHC) (MIM 602782) and sinus histiocytosis with massive lymphadenopathy (SHML), all comprising granulomatous lesions [16], [17], [18], [19], [20], [21], [22], [23] (Figure 3C). Moreover, our patients presented a few of the non-granulomatous clinical phenotypes previously reported in patients with *SLC29A3* mutations, such as insulin-dependent diabetes and finger contracture for P1 (Table 1). The c.243delA variant in *SLC29A3* is therefore most likely disease-causing in these patients.

### c.243delA leads to the expression of a mRNA splice variant of *SLC29A3* that is otherwise noncoding

The *SLC29A3* gene comprises six exons and encodes human equilibrative nucleoside transporter 3 (hENT3). Using the TA cloning technique to amplify and sequence the full-length cDNA of *SLC29A3* from control EBV-B cells, we identified three different mRNA variants. Transcript variant 1 (NM_018344.5) encodes a protein of 475 amino acids and accounted for more than 75% of the transcripts. Transcript variant 2 (NM_001174098.1) encodes a protein of 258 amino acids and accounted for less than 5% of *SLC29A3* transcripts. The final transcript variant detected in control cells was variant 3 (NR_033413.1). Variant 3 is normally a noncoding RNA, as it skips exon 3 (83 base pairs), leading to a frameshift and a premature termination codon at amino-acid position 197 (Figure 3D). The c.243delA frameshift mutation would lead to a premature termination codon at position 100 in the two coding mRNA variants (transcript variants 1 and 2). This mutation would therefore be expected to result in the nonsense-mediated mRNA decay (NMD) of these variants [24], [25]. However, the c.243delA mutation counterbalances the frameshift induced by the splicing out of exon 3 in variant 3. The combination of the deletion and the splicing out of exon 3 results in a loss of 83+1 = 84 base pairs, which is equivalent to 28 codons. This should result in a return to the correct reading frame after the first codon of exon 4, and the absence of a premature termination codon (Figure 3D). We tested this hypothesis by amplifying cDNA generated from RNA extracted from EBV-B cells and assessing the proportion of each variant in cells from controls or from the patients. For each patient and two healthy controls, we sequenced about 80 clones obtained by TA cloning. Transcript variant 3 accounted for less than 20% of the clones obtained from the controls, whereas it accounted for more than two third of the clones from the patients (Figure 3D). Total *SLC29A3* transcript levels, as assessed by quantitative PCR (Q-PCR) with a probe binding at the junction of exons 5 and 6 and recognizing both WT and mutated variants 1 and 3, were similar in cells from patients and controls (Figure 3E). Thus, the c.243delA mutation leads to an increase in both the relative proportion and the absolute quantity of *SLC29A3* transcript variant 3 in the patients' cells, and induces NMD of transcript variants 1 and 2. This situation is the opposite of that observed in cells from healthy controls.

### The mutated *SLC29A3* transcript variant 3 produces a stable hENT3 protein isoform with partial nucleoside transport activity

**Figure 4:**
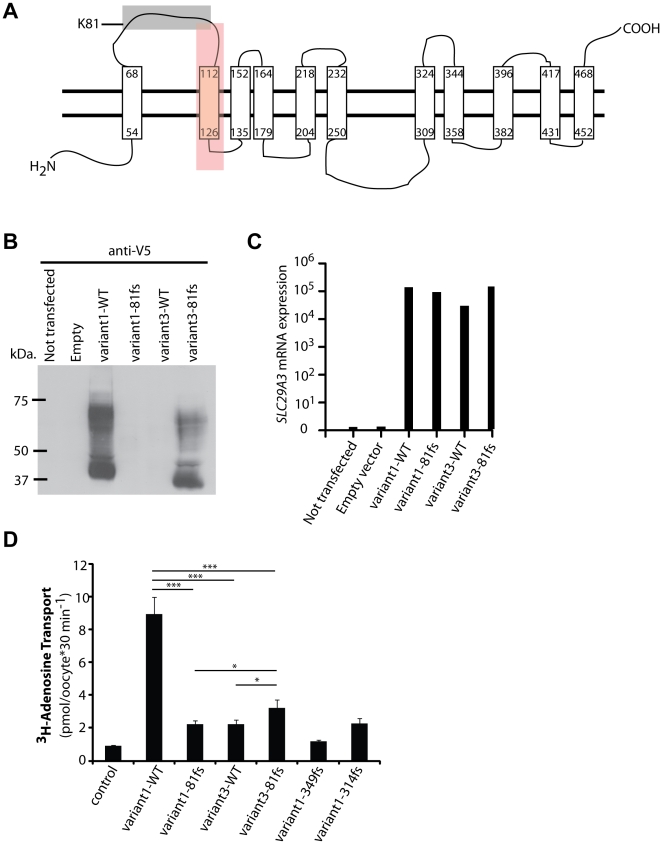
Expression and characteristics of hENT3-variant3-81fs. (A) Scheme of hENT3-variant1-WT based on the protein structure predicted by SVMtm or TMpred programs. Transmembrane domains are represented by long rectangles. The gray and red boxes highlight the amino acids that are not identical in hENT3-variant1-WT and hENT3-variant3-81fs. In the gray box: amino acids 81–100 that are replaced by 20 other amino acids in hENT3-variant3-81fs. In the red box: amino acids 101–128 that are deleted in hENT3-variant3-81fs. (B) Levels of hENT3 proteins were assessed by immunoblotting with an anti-V5-tag antibody. The V5 tag was located at the C-terminus of hENT3. (C) The graph shows the relative *SLC29A3* mRNA levels measured by Q-PCR for the same experiment with the V5-tagged constructs. Threshold cycles (Ct) for SLC29A3, normalized with respect to those of GUS (ΔCt), are plotted as 2^−(ΔCt)^. Immunoblotting and Q-PCR results representative of three independent experiments are shown. (D) Adenosine transport activity. Uptake of [^3^H]adenosine (0.026 µM) in *Xenopus* oocytes 48 h after the injection of Δ36hENT3-variant1-WT or other variants with or without the 81fs mutation as well as one H (349fs) and one PHID syndrome (314fs) mutant cRNAs. pH-dependent uptake at pH 5.5. The data shown are the means ± SEM from three independent experiments. ^*^, *p*<0.05, ^***^, *p*<0.001.

hENT3 belongs to a group of solute carrier (SLC) transporters widely conserved in eukaryotes, the ENT or SLC29 family [26], [27]. The four members of the ENT family are passive transporters, Na^+^ independent, and display a broad tissue distribution [28], [29]. hENT3 is a protein with 11 transmembrane domains, according to *in silico* predictions by SVMtm (http://ccb.imb.uq.edu.au/svmtm/) and the TMpred program (http://www.ch.embnet.org/software/TMPRED_form.html), and analogy with the topology of hENT1 [26], [30], which has a sequence 29% identical to that of hENT3 (Figure 4A). We hypothesized that the mutated transcript variant 3 would lead to the expression of a new, mutated isoform of hENT3 (possible model based on the *in silico* predictions of SVMtm or TMpred in Figure S1) and that the loss of transmembrane domain 2 would have no effect on the stability of this protein. The amino acid sequences of the mutated proteins encoded by the different transcript variants are shown in Supplementary Text S1. No commercial antibodies against hENT3 gave conclusive results in our hands. We therefore generated eight constructs carrying *SLC29A3* variant 1 WT or with the c.243delA mutation, variant 3 WT or with the c.243delA mutation with either a V5 tag at the C-terminus or a Myc tag at the N-terminus, to test our hypothesis. The transfection of HEK293T cells with these constructs demonstrated that hENT3-variant3-81fs was expressed, resulting in levels of protein similar or slightly lower to those of hENT3-variant1-WT (Figure 4B and Figure S2). The bands corresponding to hENT3-variant3-81fs showed that the hENT3-variant3-81fs proteins migrated slightly more rapidly than the hENT3-variant1-WT, due to the loss of 28 amino acids in the protein encoded by mutated variant 3 (Figure 4B). The multiple bands observed are consistent with findings for previous hENT3 blots on HeLa cells or diverse tissues [28], and may correspond to oligomers and precursor or degraded forms of the transporter in addition to hENT3. We checked that the cells were efficiently transfected, by carrying out Q-PCR to assess levels of *SLC29A*3 mRNA (Figure 4C). We demonstrated that this mutation resulted in the stable protein expression of an otherwise noncoding splice variant of *SLC29A3.*

Two members of the ENT family, hENT1 and hENT2 localize at the plasma membrane whereas hENT3 was first shown to localize in the late endosome/lysosome membrane [28] and then at the mitochondrial membrane [31], all of them significantly contributing to nucleoside and nucleobase salvage [27]. Moreover, hENT3 has been reported to be a pH-dependent intracellular transporter with maximal activity at pH 5.5 [28]. We investigated the functionality of hENT3-variant3-81fs, by assessing its adenosine transport activity at pH 5.5. As previously described [18], we first introduced the mutant and WT *SLC29A3* sequences into a *Xenopus* oocyte expression vector, for the generation of capped RNAs (cRNA) by *in vitro* transcription, which we then injected into *Xenopus* oocytes, to investigate changes in [^3^H] adenosine transports. We found that hENT3-variant3-81fs presented a highly decreased adenosine transporter activity compared to hENT3-variant1-WT (Figure 4D). However, hENT3-variant3-81fs displayed significant higher levels of transporter activity compared to hENT3-variant1-81fs or hENT3-variant3-WT, or even hENT3-variant1-314fs or hENT3-variant1-349fs, two mutants leading to PHID and H syndrome respectively [16], [19] (Figure 4D). These findings may account for the previously unreported, narrow, and milder clinical phenotype of the patients than of other patients with *SLC29A3* mutations, including frameshift deletions in particular. Three other frameshift mutations in *SLC29A3* have been reported (Figure 3C). However, the c.307delTT mutation generates an immediate termination codon [20], and both the c.940delT and c.1045delC mutations are located in exon 6 [16], [19], which encompasses the 3′-UTR. Therefore, our genetic mechanism of interest does not take place in cells carrying these mutations.

## Discussion

Mutations in the *SLC29A3* gene have been implicated in various related syndromes in humans: ‘H syndrome’ [19], [21], PHID [16], FHC and SHML [20]. Some patients may even present a combination of phenotypes from two or more of these syndromes [22], [32] (Table 1). This led to the suggestion that these phenotypes should be grouped together as ‘*SLC29A3* disorder’ [20]. The histological features of the lesions seem to be the most uniform phenotype in these patients. Doviner *et al.* proposed standardized criteria useful for diagnosis in a study of the histological features of skin biopsy specimens from 10 patients with *SLC29A3* disorder [17]. Their analysis revealed the presence of striking mononuclear infiltrates of monocyte-derived cells, consisting partly of small CD68^+^ histiocytes and of plasma cells. In addition, the small CD68^+^ histiocytes had an unusual distended endoplasmic reticulum and few lysosomes [17]. We describe here two siblings with a novel homozygous mutation in *SLC29A3* (c.243delA). Histological analysis of the nasal mucosa lesions of these patients showed characteristic widespread fibrosis and infiltrates of specific histiocytes and plasma cells typical of *SLC29A3* disorder [17]. Overall, the genetic analysis, the revised histological analyses and the fact that *Klebsiella Rhinoscleromatis* culture was negative on samples from the patients showed that the patients did not display RS. This correction of diagnosis highlights the value of WES as a diagnostic tool in patients with rare and complex phenotypes.

However, these patients had unique sets of clinical manifestations, different from that of other patients with mutations in *SLC29A3*. In particular, they displayed no cutaneous hyperpigmentation and the mucocutaneous lesions seemed to be restricted to the nasal mucosa, resulting in the initial diagnosis of RS. No hepatosplenomegaly, heart abnormalities, hearing loss or hypogonadism were detected. Thus, P1 and P2 have a mild version of *SLC29A3* disorder. Among the two patients we report, P2 had the mildest phenotype, not displaying any signs of insulin-dependent diabetes or exocrine pancreatic deficiency. On the other hand, P2 developed retroperitoneal fibrosis (RF) after 12 years of nasal infiltration and chronic inflammation. RF has never before been reported in a patient with *SLC29A3* mutation, but was frequently associated with Erdheim-Chester disease, another rare non-Langerhans cell histiocytosis of unknown origin [33]. Our findings expand the genetic and clinical spectra of *SLC29A3* disorders and illustrate how WES could rapidly expand the clinical boundaries of known genetic defects, as previously illustrated by the discovery of STIM1 deficiency in a kindred with Kaposi sarcoma (KS), which revealed both the first genetic etiology of KS and a new phenotype of STIM1 deficiency [10].

Previous studies on *SLC29A3* disorder have shown that the *SLC29A3* genotype alone cannot explain the variability in clinical presentation. For example, the p.G437R mutation was identified in patients with H syndrome, PHID, or FHC [16], [19], [20]. Moreover, two sisters carrying the same *SLC29A3* haplotype (p.G427S/G437R) displayed also two different phenotypes. The older one had a severe form of both H syndrome and PHID whereas the younger sister presented with PHID only [22]. This is consistent with the fact that P1 and P2 do not have exactly the same clinical phenotype. Yet they both present with phenotypes much milder than the majority of previously reported patients. This was unexpected, as no mild phenotype has been observed in patients with homozygous frameshift or nonsense mutations [16], [19]. The only patient with a mild phenotype had a homozygous missense mutation (p.R363Q) [23]. Mutations in *VCL* or in other genes in the linked regions, or elsewhere in the genome, might have contributed to the mild clinical phenotype. An alternative explanation is that, despite the frameshift mutation, the mutant protein might retain some function in these patients.

Indeed, we showed how this frameshift mutation, which is the first identified mutation in exon 2 of *SLC29A3*, is hypomorphic. This mutation decreases the levels of transcript variant 1 through NMD and results in an absence of the WT protein, but allows the expression of a mutated mRNA transcript variant 3, which would ordinarily be subject to NMD. This hENT3-variant3-81fs variant was expressed but displayed lower levels of adenosine transporter activity than the hENT3-variant1-WT protein. This intermediate level of transporter activity is consistent with the mild phenotype of the patients, which lies somewhere between those of healthy individuals and of other patients with more severe *SLC29A3* disorders. This is, to our knowledge, the first mutation to be characterized that leads to the expression of an mRNA variant that is normally subject to NMD. This new genetic mechanism may be general and may account for the hypomorphic nature of some frameshift mutations in other genes, those located towards the 5′ end of a gene in particular. Usually it is thought that mRNA variants carrying premature stop codons due to alternative splicing and, thus, subject to NMD are involved in gene regulation [34], [35]. We provide here an example of mRNA splice variants playing a ‘rescue’ role, leading to the production of a functional protein. This study highlights a potential new ‘rescue’ role for noncoding mRNA splice variants.

## Materials and Methods

### Ethics statement

This study was conducted in accordance with the principles of the Declaration of Helsinki. The study was approved by the Rockefeller Institutional Review Board (IRB approval number: 2010-A00650-39). All patients provided written informed consent for the collection of samples and subsequent analysis, and for the publication of pictures.

### Whole-genome linkage analysis

We genotyped the two patients (P1 and P2) and their parents (I.1 and I.2) with the Affymetrix genome-wide SNP array 6.0 (Affymetrix, Santa Clara, CA) comprising 909,622 SNPs, at the Rockefeller University Genomics Resource Center. Genotyping was highly successful, with call rates of 98 to 99% across study subjects. Only autosomal chromosome SNPs with a 100% call rate and a minor allele frequency (MAF) >0.25 among parents were retained, and no Mendelian errors were allowed, leaving 342,565 high-quality SNPs for linkage analysis. Genome-wide homozygosity mapping was performed using MERLIN [36], under a recessive model, with penetrance set to 0.9 and no allowance for sporadic cases. Multipoint LOD scores were obtained at each position, and regions longer than 1 Mb in length attaining the maximum LOD score of 1.8 for the family were defined as priority regions for the screening of exome sequencing data.

### Whole-exome sequencing

Exome capture was performed with the Agilent SureSelect Human All Exon 38 Mb kit (Agilent Technologies). Single-end sequencing was performed on an Illumina Genome Analyzer IIx (Illumina) generating 72-base reads. We aligned the sequences with the hg19 reference build of the human genome, using the Burrows-Wheeler Aligner [37]. Downstream processing and variant calling were carried out with the Genome Analysis Toolkit (GATK) [38], Samtools [39] and Picard tools (http://picard.sourceforge.net). Substitution and indel calls were made with GATK Unified Genotyper. All calls with read coverage 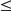
4× and a phred-scaled SNP quality of 30 were filtered out. Variants were annotated with GATK GenomicAnnotator.

### Sanger sequencing of genomic DNA

Genomic DNA was isolated from the peripheral blood mononuclear cells of the patients and members of their family. The 5′-GCTCTCCAACCAGGCTTTGG-3′ and 5′-CTGGAAATGTGTCTGAAATCTC-3′ primers were used to sequence *SLC29A3* exon 2 and its flanking intron regions.

### Quantitative RT-PCR (Q-PCR)

Total RNA from EBV-B cells was used to generate cDNA with the SuperScript III First-Strand Synthesis System (Invitrogen). *SLC29A3* expression was assessed using the *Taq*Man gene expression assay: Hs00983219_m1*, which binds to the exon 5–6 boundary. *VCL* expression was assessed using the *Taq*Man gene expression assay: Hs00419715_m1. The results obtained were normalized as a function of GUS (β-glucuronidase) gene expression.

### Analysis of the different *SLC29A3* transcripts

Full-length *SLC29A3* cDNA was amplified from the EBV-B cells of patients and controls with the 5′-CACCGACATGGCCGTTGTCTCAGAGG-3′ and 5′-GATGAGGTGCACCAGGAGGGTAGA-3′ primers. *SLC29A3* exons 2–4 were amplified from cDNA isolated from the EBV-B cells of patients and controls, with the 5′-CAGAGGACGACTTTCAGCAC-3′ and 5′-CGCTGAGGATCACCATGCAG-3′ primers. The amplicons were inserted into pCR2.1 with the TA cloning kit (Invitrogen) and sequenced with the same pair of primers. Sequences were analyzed with Lasergene software (DNASTAR).

### Mutagenesis of *SLC29A3* construct and transfection of HEK293T cells

WT variant 1 and variant 3 cDNA sequences were inserted into the pcDNA3.1 vector with a V5 tag at the C-terminus by using the pcDNA3.1/V5-His TOPO TA Expression kit from Invitrogen (#K4800-01). WT variant 1 and variant 3 cDNA sequences were inserted into pCMV6-XL4 vector (OriGene) with a Myc tag at the N-terminus (BlueHeron). The c.243delA mutation was introduced with the QuickChange site-directed mutagenesis kit from Stratagene (#200518). Primers for mutagenesis were synthesized by Integrated DNA Technologies. For c.243delA, we used the following primers: forward, 5′- AGTACTGGATGTTCAACTCCGCAACTCC-3′ and reverse, 5′- GGAGTTGCGGAGTTGAACATCCAGTACT-3′. Plasmids containing the WT or mutated sequences were then amplified and purified with the QIAprep Spin Miniprep Kit from Qiagen (#27106). The resulting plasmids were used to transfect HEK293T cells in six-well plates, using Lipofectamine LTX (Invitrogen, #15338-100).

### Western blotting

Proteins were extracted 24 hours after transfection in lysis buffer (50 nM TrisHCl, 150 mM NaCl, 1% NP-40, proteinase inhibitors) for immunoblotting. Proteins were separated by electrophoresis in a 9% acrylamide gel and blotted with, either a Myc-tag antibody (Cell Signaling, #2272) or a V5-tag antibody (Invitrogen, #R960CUS).

### Mutant hENT3 cRNA expression in *Xenopus* oocytes and measurement of transport activity

*Xenopus* oocyte expression constructs for hENT3-variant3-WT were generated by PCR with the pOX-del36hENT3 (variant1-WT) construct [18], [31] as a template. We deleted the codons corresponding to the first 36 amino acids of hENT3 from these vectors, to target the protein to the cell surface to render the experiment feasible, as previously described [18], [28]. The *Hind*III and *Xba*I restriction sites were used. The following primers were used to amplify the insert from pCMV6-XL4-Myc-hENT3-variant3-WT: forward, 5′- CGATAAGCTTCAATAATGGACCGCCCGCCCCCTGGCC-3′ and reverse, 5′- CGCGTCTAGACTAGATGAGGTGCACCAGGAGGGTAGA-3′. The inserts were then released from the pOX-del36hENT3 vector by cutting with *Hind*III and *Xba*I restriction enzymes (New England Biolabs #R0104 and #R0145). Inserts and vectors were then extracted from the gel and ligated together overnight, with T4 DNA ligase (New England Biolabs #M0202). We then used the QuickChange site-directed mutagenesis kit from Stratagene (#200518) to generate hENT3-variant1-81fs and hENT3-variant3-81fs. We used the following primers: forward, 5′- AGTACTGGATGTTCAACTCCGCAACTCC-3′ and reverse, 5′- GGAGTTGCGGAGTTGAACATCCAGTACT-3′.

We generated cRNAs and carried out expression studies in *Xenopus* oocytes as previously described [18], [31]. We injected water (control) or mutant-hENT3 cRNAs (200 ng/µl) into 80–100 defolliculated oocytes. We allowed expression of the mutant constructs to occur over a 24-hour period and then incubated viable oocytes with ^3^H-adenosine in a transport buffer containing 100 mM NaCl, 2 mM KCl, 1 mM CaCl_2_, 1 mM MgCl_2_, and 10 mM HEPES, pH 5.5. The rate of adenosine influx rates into oocytes over a period of 30 minutes was then determined by liquid scintillation counting and plotted.

### Statistical analysis

Student's *t* test was used to identify significant differences, and experiments were repeated three times.

## Supporting Information

Figure S1 **Predicted model of hENT3-variant3-81fs.** Scheme of hENT3-variant3-81fs based on the protein structure predicted by SVMtm or TMpred programs. Transmembrane domains are represented by long rectangles.
(TIF)

Figure S2 **hENT3-variant3-81fs leads to a stable protein expression.** Levels of hENT3 proteins were assessed by immunoblotting with an anti-Myc-tag antibody. The Myc tag was located at the N-terminus part of the protein. The immunoblot is representative of three independent experiments.
(TIF)

Table S1 **Variants identified by WES in the linked intervals for P1.**
(DOC)

Text S1 **Predicted hENT3 WT and mutant sequences for all variants.** Alternate blue and black colors indicate the different exons of the gene. Amino acids in red are encoded by a codon overlapping two exons. A space was introduced each 10 amino acids for the sake of clarity.
(DOC)
