# Supplementary figures and images for: A Mild Form of SLC29A3 Disorder: A Frameshift Deletion Leads to the Paradoxical Translation of an Otherwise Noncoding mRNA Splice Variant
Source: PLoS One. 2012 Jan 4;7(1):e29708. doi: 10.1371/journal.pone.0029708 (PMC3251605; doi:10.1371/journal.pone.0029708)

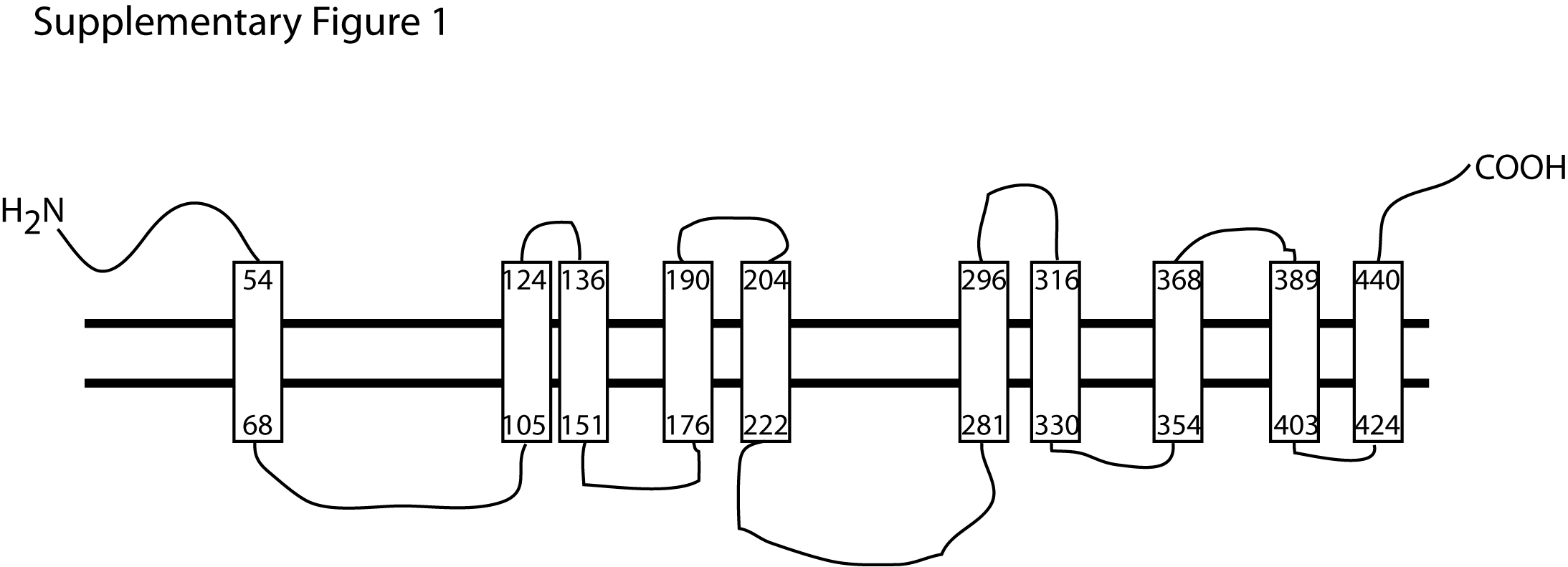

Supplement: Figure S1 — Predicted model of hENT3-variant3-81fs. Scheme of hENT3-variant3-81fs based on the protein structure predicted by SVMtm or TMpred programs. Transmembrane domains are represented by long rectangles. (TIF) [file pone.0029708.s001.tif]

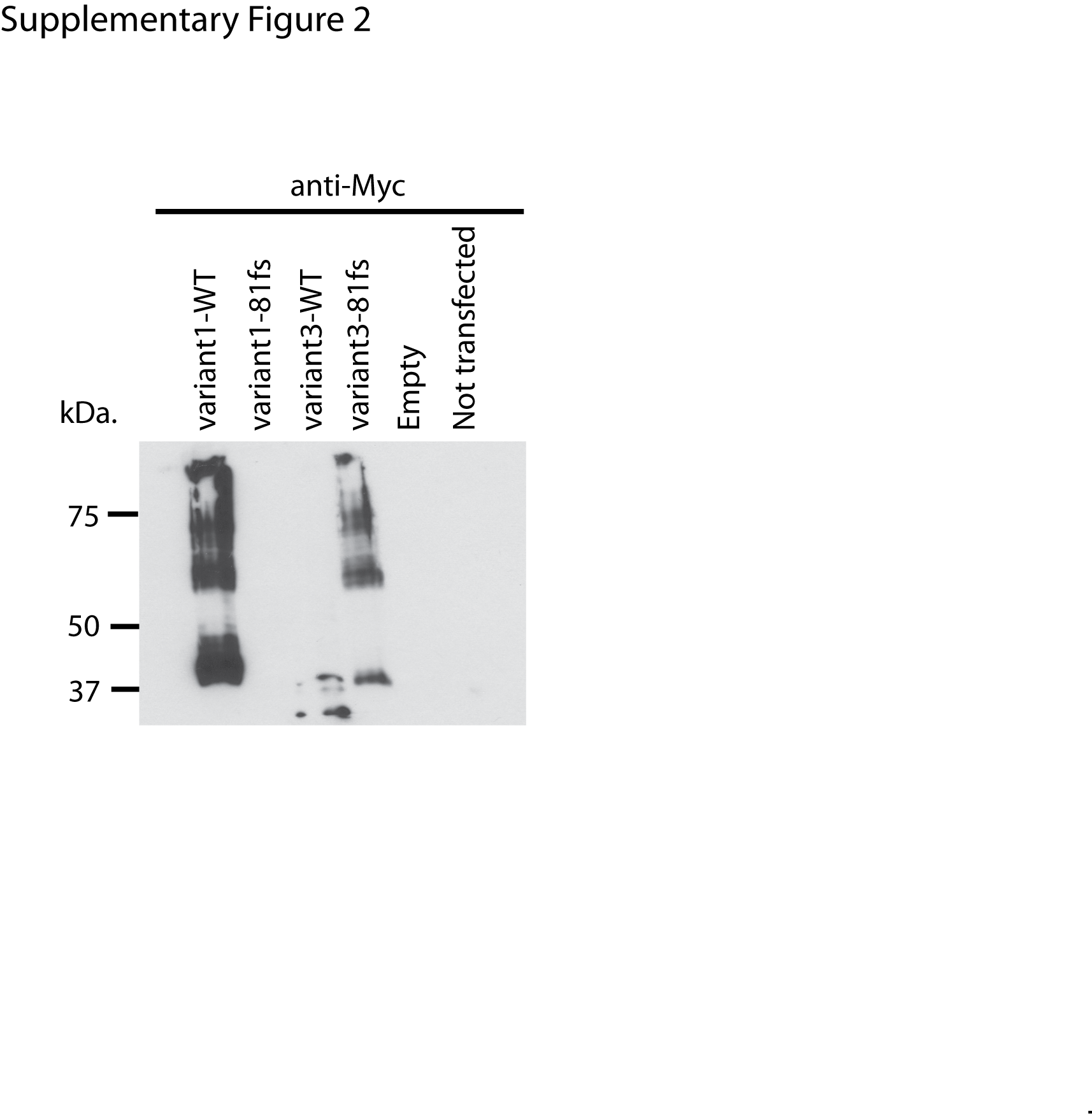

Supplement: Figure S2 — hENT3-variant3-81fs leads to a stable protein expression. Levels of hENT3 proteins were assessed by immunoblotting with an anti-Myc-tag antibody. The Myc tag was located at the N-terminus part of the protein. The immunoblot is representative of three independent experiments. (TIF) [file pone.0029708.s002.tif]
