# Supplementary material for: A Mild Form of SLC29A3 Disorder: A Frameshift Deletion Leads to the Paradoxical Translation of an Otherwise Noncoding mRNA Splice Variant
Source: PLoS One. 2012 Jan 4;7(1):e29708. doi: 10.1371/journal.pone.0029708 (PMC3251605; doi:10.1371/journal.pone.0029708)
Supplement: Table S1 — Variants identified by WES in the linked intervals for P1. (DOC) [file pone.0029708.s003.doc]

**Table S1: Variants identified by WES in the linked intervals for P1.**

| Chr. | Position^a^ | Allele | 1kg.AF^b^ | dbsnp.ID^c^ | in hgid^d^ | gene | strand | Functional impact |
| --- | --- | --- | --- | --- | --- | --- | --- | --- |
| 1 | 166039898 | C/G | 0.2765 | rs41269662 | yes | *FAM78B* | - | silent |
| 1 | 167095163 | G/C | 0.756 | rs267745 | yes | *DUSP27* | + | missense (p.E265D) |
| 1 | 167095765 | G/A | 0.5766 | rs6668826 | yes | *DUSP27* | + | missense (p.R466H) |
| 1 | 167095881 | G/A | 0.5303 | rs3795605 | yes | *DUSP27* | + | missense (p.A505T) |
| 1 | 167097739 | C/A | 0.6118 | rs2281959 | yes | *DUSP27* | + | missense (p.T1124N) |
| 1 | 167815030 | A/G | 0.1633 | rs12048517 | yes | *ADCY10* | - | silent |
| 1 | 167817639 | A/G | 0.7481 | rs203795 | yes | *ADCY10* | - | silent |
| 1 | 167825485 | T/C | 0.6132 | rs2071921 | yes | *ADCY10* | - | missense (p.I605V) |
| 1 | 167825606 | A/G | 0.3668 | rs2071922 | yes | *ADCY10* | - | silent |
| 1 | 167849414 | A/G | 0.47753 | rs203849 | yes | *ADCY10* | - | silent |
| 1 | 167906239 | A/C | 1 | rs203788 | yes | *DCAF6* | + | splice |
| 1 | 168014204 | T/A | 0.09 | rs41271647 | yes | *DCAF6* | + | missense (p.589H) |
| 1 | 168698173 | C/T | 0.44402 | rs1052591 | yes | *DPT* | - | silent |
| 1 | 169390957 | A/T | 0.31532 | rs35107735 | yes | *C1orf114* | - | missense (p.F238I) |
| 1 | 169391154 | A/G | 0.845 | rs3820059 | yes | *C1orf114* | - | missense (p.F172S) |
| 1 | 169498975 | T/C | 0.36496 | rs6030 | yes | *F5* | - | missense (p.M1769V) |
| 1 | 169500210 | T/C | 0.1332 | rs6010 | yes | *F5* | - | silent |
| 1 | 169510118 | G/A | 0.1264 | rs9332608 | yes | *F5* | - | missense (p.P1409S) |
| 1 | 169510380 | G/A | 0.27239 | rs9287090 | yes | *F5* | - | silent |
| 1 | 169510524 | A/G | 0.29077 | rs1800594 | yes | *F5* | - | silent |
| 1 | 169511555 | T/C | 0.28102 | rs6032 | yes | *F5* | - | missense (p.K930E) |
| 1 | 169511734 | T/C | 0.28597 | rs4525 | yes | *F5* | - | missense (p.H870R) |
| 1 | 169511755 | T/C | 0.28494 | rs4524 | yes | *F5* | - | missense (p.K863R) |
| 1 | 169512027 | T/C | 0.28895 | rs6021 | yes | *F5* | - | silent |
| 1 | 169512093 | A/G | 0.28947 | rs6017 | yes | *F5* | - | silent |
| 1 | 169512120 | G/A | 0.28165 | rs6016 | yes | *F5* | - | silent |
| 1 | 169519049 | T/C | 0.98626 | rs6025 | yes | *F5* | - | missense (p.Q534R) |
| 1 | 169566313 | C/T | 0.8482 | rs6127 | yes | *SELP* | - | missense (p.D603N) |
| 1 | 169823660 | C/T | 0.24638 | rs1062976 | yes | *SCYL3* | - | silent |
| 1 | 169823718 | T/C | 0.60967 | rs4656197 | yes | *SCYL3* | - | missense (p.Q621R) |
| 1 | 169951997 | C/T | 0.4238 | rs33943686 | yes | *KIFAP3* | - | silent |
| 1 | 170928637 | T/C | 0.0014 | 0 | yes | *C1orf129* | + | silent |
| 1 | 170934394 | T/C | 0.06781 | rs16863872 | yes | *C1orf129* | + | missense (p.Y160H) & splice |
| 1 | 170959029 | C/T | 0.3475 | rs16863922 | yes | *C1orf129* | + | silent |
| 1 | 170959088 | C/T | 0.3958 | rs28634500 | yes | *C1orf129* | + | silent |
| 1 | 171154959 | A/G | 0.0958 | rs2020870 | yes | *FMO2* | + | missense (p.D36G) |
| 1 | 171168584 | C/T | 0.4487 | rs2020862 | yes | *FMO2* | + | missense (p.S195L) |
| 1 | 171168585 | A/G | 0.532 | rs2020861 | yes | *FMO2* | + | silent |
| 1 | 171679958 | G/A | 0.4564 | rs10913530 | yes | *VAMP4* | - | splice |
| 1 | 193051685 | G/A | 0.67156 | rs2275444 | yes | *TROVE2* | + | splice |
| 1 | 196227526 | A/G | 0.99903 | rs977157 | yes | *KCNT2* | - | silent |
| 1 | 196309523 | C/T | 0.05104 | rs142546287 | yes | *KCNT2* | - | silent |
| 1 | 196654324 | A/C | 0.8337 | rs1061147 | yes | *CFH* | + | silent |
| 1 | 196659237 | C/T | 0.8345 | rs1061170 | yes | *CFH* | + | missense (p.H338Y) |
| 1 | 196695742 | A/G | 0.3666 | rs3753396 | yes | *CFH* | + | silent |
| 1 | 196709774 | G/T | 0.3684 | rs1065489 | yes | *CFH* | + | missense (p.E936D) |
| 1 | 196799691 | G/A | 0.127 | rs12406079 | yes | *CFHR1* | + | silent |
| 1 | 196801042 | G/T | 0.5212 | rs4230 | yes | *CFHR1* | + | silent |
| 1 | 196801078 | A/T | 0.4878 | rs414628 | yes | *CFHR1* | + | silent |
| 1 | 196884258 | A/T | 0.1065 | rs150845796 | yes | *CFHR4* | + | silent |
| 1 | 197030201 | T/C | 0.90971 | rs5997 | yes | *F13B* | - | splice |
| 1 | 197031021 | C/T | 0.91236 | rs6003 | yes | *F13B* | - | missense (p.R115H) |
| 1 | 197061086 | A/C | 0.0348 | rs36004306 | yes | *ASPM* | - | missense (p.L1547R) |
| 1 | 197070442 | G/T | 0.36496 | rs3762271 | yes | *ASPM* | - | missense (p.L633I) |
| 1 | 197070697 | T/C | 0.3475 | rs41310927 | yes | *ASPM* | - | missense (p.S548G) |
| 1 | 197070707 | G/A | 0.36765 | rs41308365 | yes | *ASPM* | - | silent |
| 1 | 197070815 | T/C | 0.91171 | rs1412640 | yes | *ASPM* | - | silent |
| 1 | 197070901 | A/G | 0.99455 | rs964201 | yes | *ASPM* | - | missense (p.Y480H) |
| 1 | 197072420 | T/C | 0.36618 | rs41310925 | yes | *ASPM* | - | silent |
| 1 | 197073932 | T/C | 0.36178 | rs2878749 | yes | *ASPM* | - | silent |
| 1 | 197091537 | A/T | 0.91637 | rs4915337 | yes | *ASPM* | - | silent |
| 1 | 197112374 | T/C | 0 | 0 | yes | *ASPM* | - | silent |
| 1 | 197112533 | G/A | 0.91481 | rs6677082 | yes | *ASPM* | - | silent |
| 1 | 197390368 | A/G | 0.99237 | rs3902057 | yes | *CRB1* | + | silent |
| 1 | 197480927 | C/T | 0.27632 | rs12142127 | yes | *DENND1B* | - | silent |
| 1 | 197480986 | C/T | 0.07743 | rs10494755 | yes | *DENND1B* | - | missense (p.G203S) |
| 1 | 197896728 | T/C | 0.3747 | rs12046958 | yes | *LHX9* | + | splice |
| 1 | 200017586 | C/G | 0.3983 | rs2821368 | yes | *NR5A2* | + | silent |
| 1 | 200618238 | T/C | 0.95472 | rs6665604 | yes | *DDX59* | - | silent |
| 1 | 200776641 | C/T | 0.2753 | rs7552353 | yes | *CAMSAP1L1* | + | silent |
| 1 | 200867552 | C/T | 0.0914 | rs2271017 | yes | *C1orf106* | + | silent |
| 1 | 200880869 | A/C | 0.1045 | rs45547233 | yes | *C1orf106* | + | missense (p.R501S) |
| 1 | 200956288 | A/G | 0.3297 | rs2275485 | yes | *KIF21B* | - | silent |
| 1 | 200959302 | G/A | 0.3473 | rs2297911 | yes | *KIF21B* | - | silent |
| 1 | 201009182 | A/G | 0.4167 | rs12139527 | yes | *CACNA1S* | - | missense (p.1800S) |
| 1 | 201047062 | G/A | 0.2391 | rs4915476 | yes | *CACNA1S* | - | silent |
| 1 | 201047075 | A/G | 0.6597 | rs4915477 | yes | *CACNA1S* | - | silent |
| 1 | 201112981 | A/G | 0.76075 | rs8158 | yes | *TMEM9* | - | silent |
| 1 | 201187764 | T/C | 0.8456 | rs10920144 | yes | *IGFN1* | + | silent |
| 1 | 201190732 | G/A | 0.2467 | rs3738269 | yes | *IGFN1* | + | silent |
| 1 | 201252866 | C/T | 0.3116 | rs2268147 | yes | *PKP1* | + | silent |
| 1 | 201282573 | A/G | 0.2062 | rs35507614 | yes | *PKP1* | + | missense (p.I196V) |
| 1 | 201285759 | A/G | 0.99712 | rs1779297 | yes | *PKP1* | + | silent |
| 1 | 201334382 | G/A | 0.76647 | rs3729547 | yes | *TNNT2* | - | silent |
| 1 | 201334795 | C/T | 0.1364 | rs3729845 | yes | *TNNT2* | - | splice |
| 1 | 201355522 | T/C | 0.7246 | rs4128458 | yes | *LAD1* | - | missense (p.K337E) |
| 1 | 201355669 | T/C | 0.0882 | rs68021059 | yes | *LAD1* | - | missense (p.T288A) |
| 1 | 201355943 | C/T | 0.43996 | rs2799677 | yes | *LAD1* | - | silent |
| 1 | 201934578 | G/A | 0.08333 | rs4648 | yes | *TIMM17A* | + | missense (p.V113I) |
| 1 | 201969082 | G/A | 0.43431 | rs1130790 | yes | *RNPEP* | + | silent |
| 1 | 201970562 | T/C | 0.99908 | rs4332408 | yes | *RNPEP* | + | silent |
| 1 | 201981218 | C/G | 0.4396 | rs11543979 | yes | *ELF3* | + | silent |
| 6 | 127768472 | G/A | 0.488 | rs2236026 | yes | *KIAA0408* | - | missense (p.S331L) |
| 6 | 127797179 | G/A | 0.5851 | rs3734448 | yes | *C6orf174* | - | silent |
| 6 | 127797374 | G/A | 0.57 | rs3734450 | yes | *C6orf174* | - | silent |
| 6 | 128388799 | G/T | 0.39515 | rs35030557 | yes | *PTPRK* | - | silent |
| 6 | 128403745 | A/G | 0.64048 | rs17828130 | yes | *PTPRK* | - | silent |
| 6 | 129381026 | C/A | 0.97615 | rs4404787 | yes | *LAMA2* | + | silent |
| 6 | 129762112 | G/A | 0.2287 | rs2297738 | yes | *LAMA2* | + | silent |
| 6 | 130152479 | T/C | 0.55712 | rs7758540 | yes | *C6orf191* | - | silent |
| 6 | 130152520 | A/C | 0.38469 | rs7776426 | yes | *C6orf191* | - | missense (p.F111V) |
| 6 | 130154686 | A/G | 0.68293 | rs4629709 | yes | *C6orf191* | - | missense (p.F80L) |
| 6 | 130374102 | C/A | 0.63254 | rs9388768 | yes | *L3MBTL3* | + | missense (p.T183N) |
| 6 | 130381246 | T/C | 0.67093 | rs7451021 | yes | *L3MBTL3* | + | silent |
| 6 | 130761804 | C/T | 0.4221 | rs12200105 | yes | *TMEM200A* | + | silent |
| 10 | 63170292 | A/G | 0.21996 | rs7083475 | yes | *TMEM26* | - | silent |
| 10 | 63520698 | T/C | 0.81148 | rs1992625 | yes | *C10orf107* | + | silent |
| 10 | 63964653 | C/T | 0.2202 | rs41274060 | yes | *RTKN2* | - | silent |
| 10 | 63995983 | C/T | 0.86464 | rs3852448 | yes | *RTKN2* | - | silent |
| 10 | 64136072 | G/C | 0.0298 | rs79142251 | yes | *ZNF365* | + | missense (p.L40F) |
| 10 | 64415184 | A/G | 0.75558 | rs7076156 | yes | *ZNF365* | + | missense (p.T62A) |
| 10 | 64573771 | C/T | 0.9956 | rs224083 | yes | *EGR2* | - | silent |
| 10 | 64927823 | C/G | 0.43611 | rs1935 | yes | *JMJD1C* | - | missense (p.E2535D) |
| 10 | 64945364 | G/A | 0.7271 | rs3211105 | yes | *JMJD1C* | - | silent |
| 10 | 64967445 | A/T | 0.9991 | rs1904294 | yes | *JMJD1C* | - | silent |
| 10 | 64974537 | A/T | 0.70236 | rs10761725 | yes | *JMJD1C* | - | missense (p.S245T) |
| 10 | 68040325 | C/T | 0.38991 | rs4548513 | yes | *CTNNA3* | - | missense (p.S596N) |
| 10 | 69571336 | G/A | 0.264 | rs3740049 | yes | *DNAJC12* | - | silent |
| 10 | 69666598 | T/C | 0.2319 | rs2273773 | yes | *SIRT1* | + | silent |
| 10 | 69773841 | G/A | 0.6607 | rs866255 | yes | *HERC4* | - | silent |
| 10 | 70051928 | A/G | 0.79035 | rs10823171 | yes | *PBLD* | - | silent |
| 10 | 70105560 | G/A | 0.84727 | rs1162753 | yes | *RUFY2* | - | silent |
| 10 | 70405855 | A/G | 0.79295 | rs3998860 | yes | *TET1* | + | missense (p.I1123M) |
| 10 | 70502288 | G/A | 0.88251 | rs1300253 | yes | *CCAR1* | + | silent |
| 10 | 70549579 | G/A | 0.06105 | rs11594683 | yes | *CCAR1* | + | silent |
| 10 | 70641860 | T/C | 0.7572 | rs1341667 | yes | *STOX1* | + | missense (p.Y153H) & splice |
| 10 | 70696713 | C/T | 0.9159 | rs5030895 | yes | *DDX50* | + | silent |
| 10 | 70700944 | A/G | 0.7667 | rs5030900 | yes | *DDX50* | + | splice |
| 10 | 70748784 | G/A | 0.4672 | rs2255607 | yes | *KIAA1279* | + | missense (p.G66S) |
| 10 | 71018660 | T/C | 0.99906 | rs1111335 | yes | *HKDC1* | + | missense (p.W721R) |
| 10 | 71026510 | C/A | 0.5158 | rs906219 | yes | *HKDC1* | + | missense (p.N917K) |
| 10 | 71060610 | A/G | 0.91236 | rs906220 | yes | *HK1* | + | missense (p.H7R) & splice |
| 10 | 71142420 | G/A | 0.7327 | rs748235 | yes | *HK1* | + | silent |
| 10 | 71168685 | A/T | 1 | rs55953810 | yes | *TACR2* | - | missense (p.M245K) & splice |
| 10 | 71391538 | T/C | 0.18563 | rs1381932 | yes | *C10orf35* | + | silent |
| 10 | 71392557 | C/T | 0.7086 | rs12020 | yes | *C10orf35* | + | silent |
| 10 | 71392692 | T/C | 0.8333 | rs1052152 | yes | *C10orf35* | + | silent |
| 10 | 71876382 | C/T | 0.76786 | rs2394656 | yes | *AIFM2* | - | splice |
| 10 | 71906150 | T/C | 0.5168 | rs4746970 | yes | *TYSND1* | - | missense (p.T65A) |
| 10 | 72300891 | G/A | 0.0067 | rs61747986 | yes | *KIAA1274* | + | missense (p.A648T) |
| 10 | 72324143 | A/G | 0.9968 | rs7093516 | yes | *KIAA1274* | + | silent |
| 10 | 72358577 | G/A | 0.6046 | rs885822 | yes | *PRF1* | - | silent |
| 10 | 72358655 | G/A | 0.1734 | rs885821 | yes | *PRF1* | - | silent |
| 10 | 72513635 | C/A | 0.2412 | rs12774070 | yes | *ADAMTS14* | + | missense (p.L937M) |
| 10 | 72513682 | C/T | 0.5692 | rs2587475 | yes | *ADAMTS14* | + | silent |
| 10 | 72520259 | C/T | 0.0908 | rs61573157 | yes | *ADAMTS14* | + | missense (p.1108S) |
| 10 | 72535007 | C/T | 0.83721 | rs2254174 | yes | *C10orf27* | - | missense (p.R237Q) |
| 10 | 72614524 | A/G | 0.04348 | rs11597050 | yes | *SGPL1* | + | silent |
| 10 | 72619205 | C/T | 0.99812 | rs827255 | yes | *SGPL1* | + | silent |
| 10 | 73039636 | G/A | 0.0125 | rs116787916 | yes | *UNC5B* | + | silent |
| 10 | 73082563 | A/G | 0.58 | rs2277257 | yes | *SLC29A3* | + | missense (p.R18G) |
| 10 | 73082751 | CA/C | 0 | 0 | no | *SLC29A3* | + | frameshift deletion (p.K81Nfs) |
| 10 | 73115941 | T/C | 0.8049 | rs2252997 | yes | *SLC29A3* | + | silent |
| 10 | 73115942 | G/A | 0.8035 | rs2252996 | yes | *SLC29A3* | + | missense (p.V239I) |
| 10 | 73121913 | A/G | 0.7958 | rs2487068 | yes | *SLC29A3* | + | missense (p.I326V) |
| 10 | 73121945 | T/C | 0.828 | rs1084004 | yes | *SLC29A3* | + | silent |
| 10 | 73520632 | A/C | 0.86692 | rs3747869 | yes | *C10orf54* | - | missense (p.D183E) & splice |
| 10 | 73521371 | C/T | 0.2464 | rs3747867 | yes | *C10orf54* | - | silent |
| 10 | 75832598 | G/C | 0 | 0 | no | *VCL* | + | missense (p.V204L) |
| 10 | 75865065 | G/A | 0.56415 | rs767809 | yes | *VCL* | + | silent |
| 10 | 75871735 | C/G | 0.7279 | rs2131956 | yes | *VCL* | + | silent |
| 10 | 76854564 | C/T | 0.8096 | rs3088142 | yes | *DUSP13* | - | missense (p.C156Y) |
| 10 | 76855412 | G/A | 0.5344 | rs3740317 | yes | *DUSP13* | - | silent |
| 10 | 76861680 | T/C | 0.7608 | rs6480771 | yes | *DUSP13* | - | missense (p.S75G) |
| 10 | 79566632 | G/A | 0.28586 | rs1058198 | yes | *DLG5* | - | silent |
| 10 | 79581401 | C/T | 0 | 0 | yes | *DLG5* | - | nonsense (p.W947X) |
| 10 | 79584178 | G/C | 0.65283 | rs1248629 | yes | *DLG5* | - | silent |
| 10 | 79616605 | T/C | 0.909 | rs1248696 | yes | *DLG5* | - | missense (p.Q140R) |
| 12 | 94645255 | A/G | 0.7124 | rs2230757 | yes | *PLXNC1* | + | silent |
| 12 | 94673277 | G/A | 0.1613 | rs7952864 | yes | *PLXNC1* | + | silent |
| 12 | 94761701 | T/C | 0.12177 | rs12426243 | yes | *CCDC41* | - | silent |
| 12 | 94769758 | A/G | 0.8805 | rs4761611 | yes | *CCDC41* | - | silent |
| 22 | 46929692 | A/G | 0.9497 | rs4823561 | yes | *CELSR1* | - | missense (p.C1126R) |
| 22 | 46931077 | G/C | 0.961 | rs4823850 | yes | *CELSR1* | - | missense (p.S664W) |
| 22 | 46931793 | G/C | 0.9571 | rs1009154 | yes | *CELSR1* | - | silent |
| 22 | 46931838 | G/A | 0.956 | rs1009155 | yes | *CELSR1* | - | silent |
| 22 | 47058992 | T/C | 1 | rs2542040 | yes | *GRAMD4* | + | silent |

a: Position coordinates correspond to the hg19, NCBI build 37

b: Frequency of the allele in the 1000 Genomes database (June 2011 release).

c: NCBI dbSNP134 ID. If present in the databse, the variant has an ID. 0 indicates the variant wasnot found in the databse.

d: Presence or not in the exomes sequenced by the Human Genetics of Infectious Diseases laboratory.
