# Supplementary material for: A Mild Form of SLC29A3 Disorder: A Frameshift Deletion Leads to the Paradoxical Translation of an Otherwise Noncoding mRNA Splice Variant
Source: PLoS One. 2012 Jan 4;7(1):e29708. doi: 10.1371/journal.pone.0029708 (PMC3251605; doi:10.1371/journal.pone.0029708)
Supplement: Text S1 — Predicted hENT3 WT and mutant sequences for all variants. Alternate blue and black colors indicate the different exons of the gene. Amino acids in red are encoded by a codon overlapping two exons. A space was introduced each 10 amino acids for the sake of clarity. (DOC) [file pone.0029708.s004.doc]

**Supplementary Text S1:** Predicted hENT3 WT and mutant sequences for all variants.

**hENT3-variant1-WT:** 475 amino acids.

MAVVSEDDFQ HSSNSTYRTT SSSLRADQEA LLEKLLDRPP PGLQRPEDRF CGTYIIFFSL GIGSLLPWNF FITAKEYWMF KLRNSSSPAT GEDPEGSDIL NYFESYLAVA STVPSMLCLV ANFLLVNRVA VHIRVLASLT VILAIFMVIT ALVKVDTSSW TRGFFAVTIV CMVILSGAST VFSSSIYGMT GSFPMRNSQA LISGGAMGGT VSAVASLVDL AASSDVRNSA LAFFLTATVF LVLCMGLYLL LSRLEYARYY MRPVLAAHVF SGEEELPQDS LSAPSVASRF IDSHTPPLRP ILKKTASLGF CVTYVFFITS LIYPAICTNI ESLNKGSGSL WTTKFFIPLT TFLLYNFADL CGRQLTAWIQ VPGPNSKALP GFVLLRTCLI PLFVLCNYQP RVHLKTVVFQ SDVYPALLSS LLGLSNGYLS TLALLYGPKI VPRELAEATG VVMSFYVCLG LTLGSACSTL LVHLI

**hENT3-variant2-WT:** 258 amino acids.

MAVVSEDDFQ HSSNSTYRTT SSSLRADQEA LLEKLLDRPP PGLQRPEDRF CGTYIIFFSL GIGSLLPWNF FITAKEYWMF KLRNSSSPAT GEDPEGSDIL NYFESYLAVA STVPSMLCLV ANFLLVNRVA VHIRVLASLT VILAIFMVIT ALVKVDTSSW TRGFFAVTIV CMVILSGAST VFSSSIYGMT GSFPMRNSQA LISGGAMGGT VSAVASLVDL AASSDVRNSA LAFFLTATVF LVLCMGLYLL LSRLEYAR

**hENT3-variant3-WT:** 196 amino acids.

MAVVSEDDFQ HSSNSTYRTT SSSLRADQEA LLEKLLDRPP PGLQRPEDRF CGTYIIFFSL GIGSLLPWNF FITAKEYWMF KLRNSSSPAT GEDPEGSDIL [G](http://ca.expasy.org/cgi-bin/dna_sequences?/work/expasy/tmp/http/seqdna.14061,1,1)CSPHPCPGL TDGHPGHLHG DNCTGEGGHF LLDPWLFCGH HCLHGDPQRC LHCLQQQHLR HDRLLSYEEL PGTDIRRSHG RDGQRRGLIG GLGCIQ

**hENT3-variant1-81fs:** 99 amino acids.

MAVVSEDDFQ HSSNSTYRTT SSSLRADQEA LLEKLLDRPP PGLQRPEDRF CGTYIIFFSL GIGSLLPWNF FITAKEYWMF NSATPPAQPP GRTLRAQTS

**hENT3-variant2-81fs:** 99 amino acids.

MAVVSEDDFQ HSSNSTYRTT SSSLRADQEA LLEKLLDRPP PGLQRPEDRF CGTYIIFFSL GIGSLLPWNF FITAKEYWMF NSATPPAQPP GRTLRAQTS

**hENT3-variant3-81fs:** 447 amino acids.

MAVVSEDDFQ HSSNSTYRTT SSSLRADQEA LLEKLLDRPP PGLQRPEDRF CGTYIIFFSL GIGSLLPWNF FITAKEYWMF NSATPPAQPP GRTLRAQTSW VAVHIRVLAS LTVILAIFMV ITALVKVDTS SWTRGFFAVT IVCMVILSGA STVFSSSIYG MTGSFPMRNS QALISGGAMG GTVSAVASLV DLAASSDVRN SALAFFLTAT VFLVLCMGLY LLLSRLEYAR YYMRPVLAAH VFSGEEELPQ DSLSAPSVAS RFIDSHTPPL RPILKKTASL GFCVTYVFFI TSLIYPAICT NIESLNKGSG SLWTTKFFIP LTTFLLYNFA DLCGRQLTAW IQVPGPNSKA LPGFVLLRTC LIPLFVLCNY QPRVHLKTVV FQSDVYPALL SSLLGLSNGY LSTLALLYGP KIVPRELAEA TGVVMSFYVC LGLTLGSACS TLLVHLI
